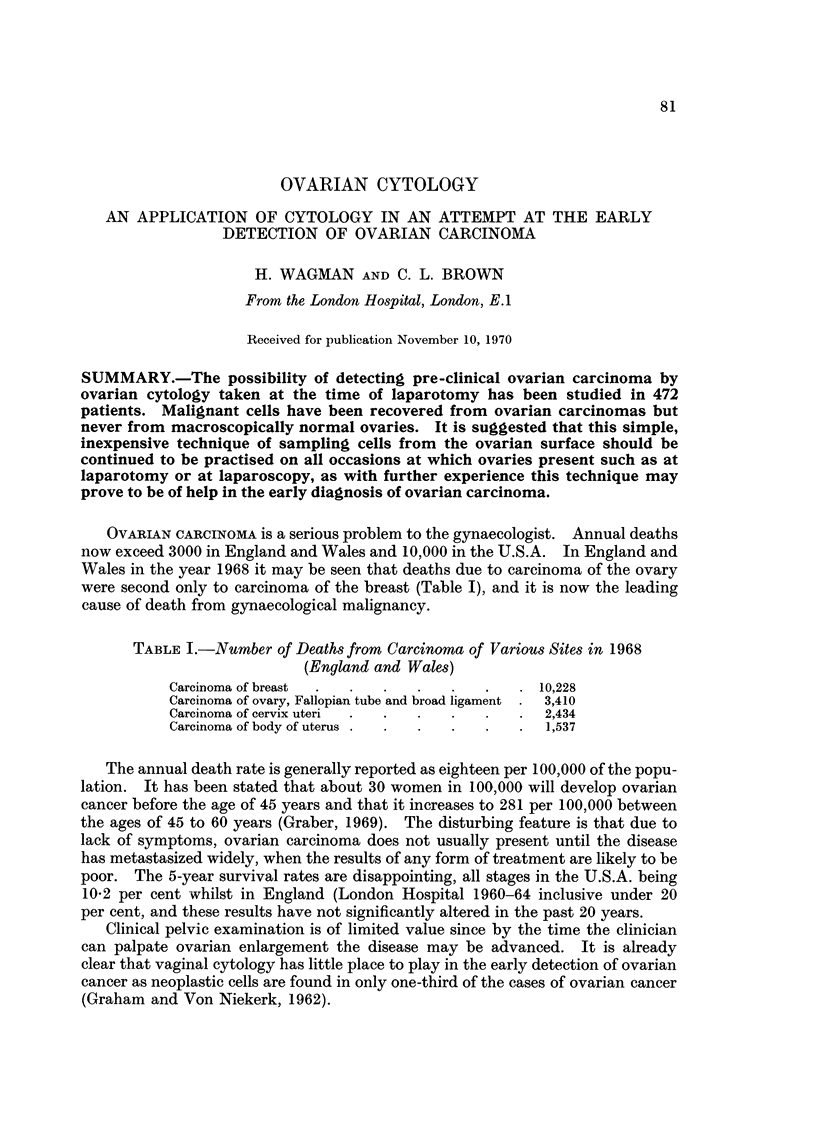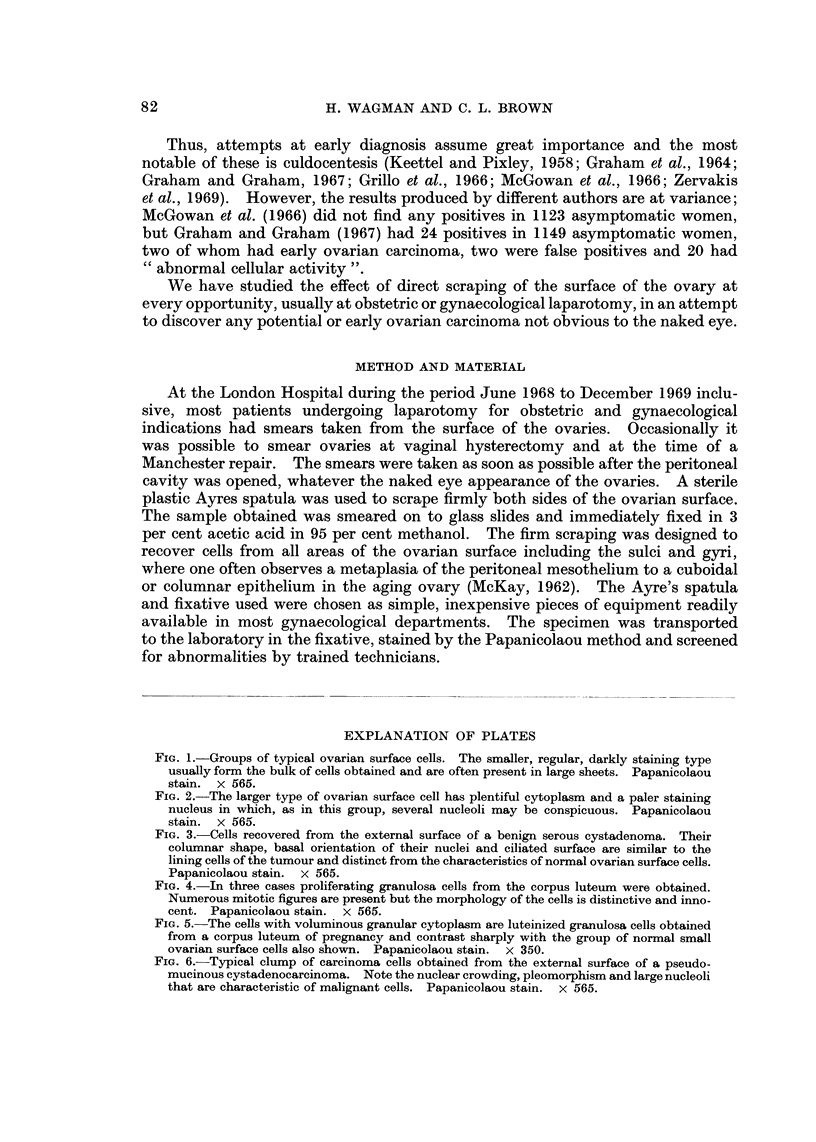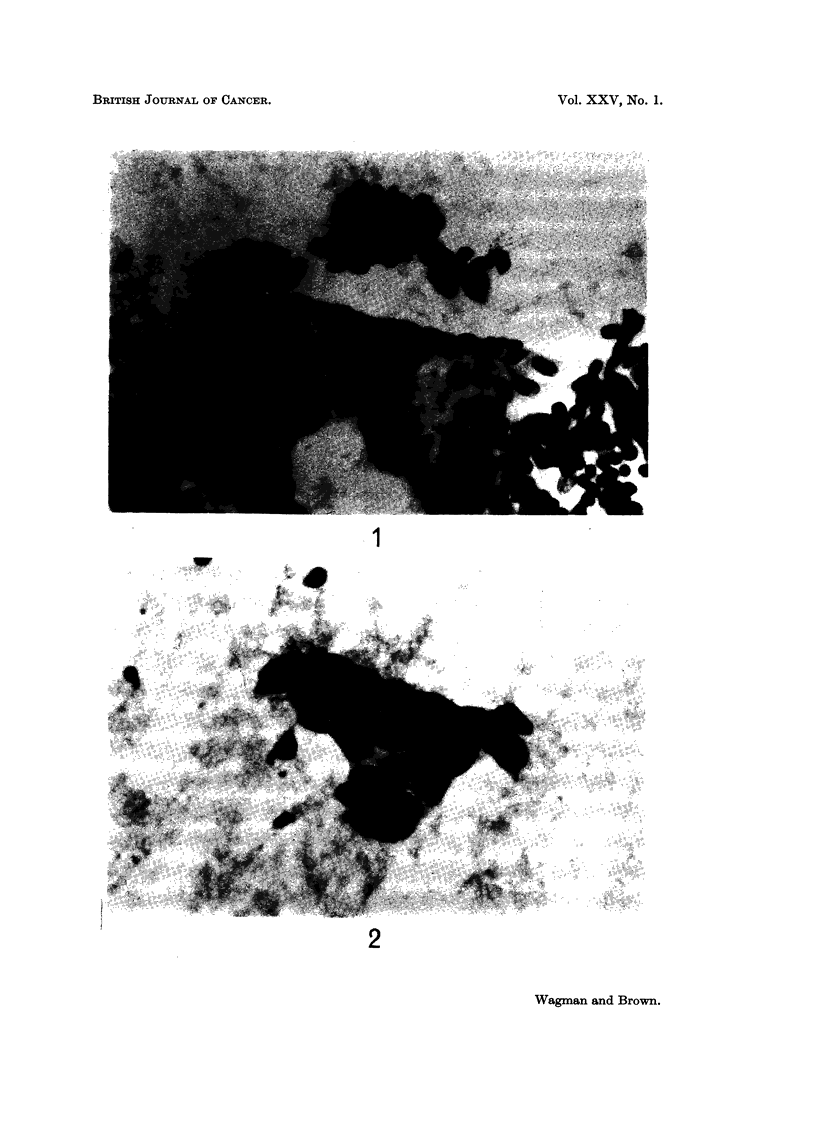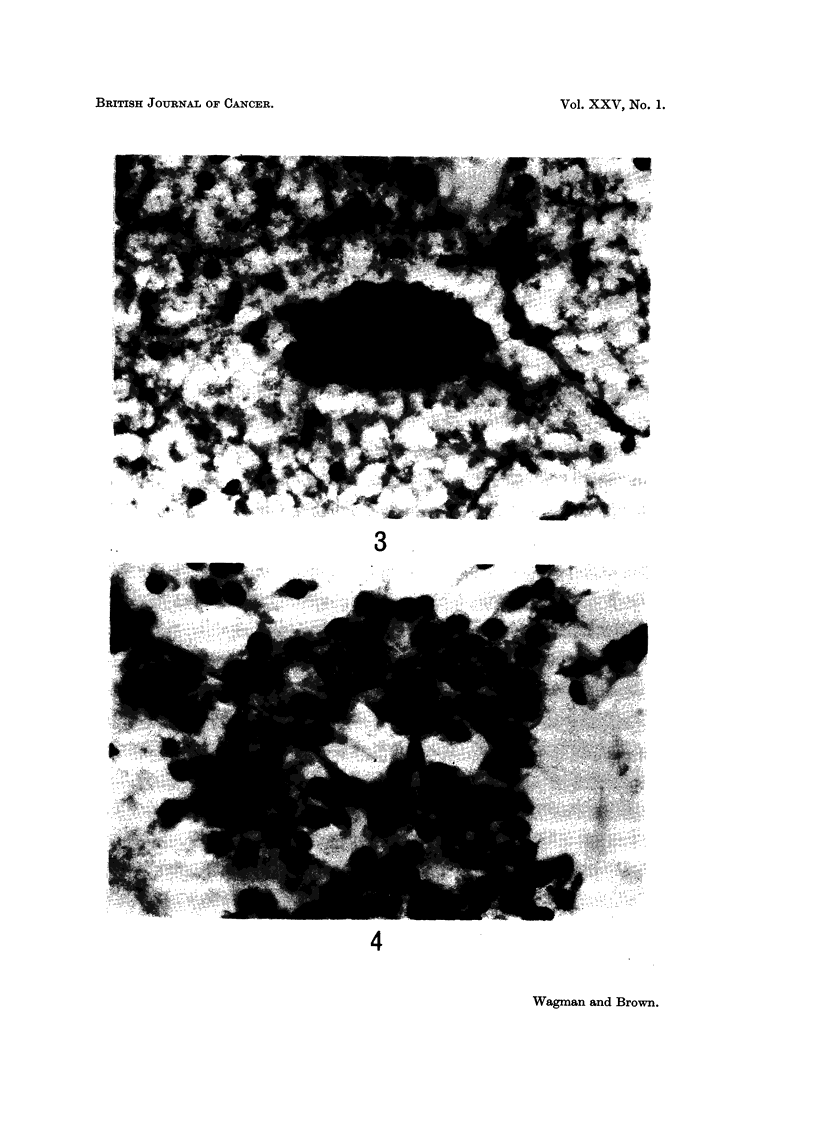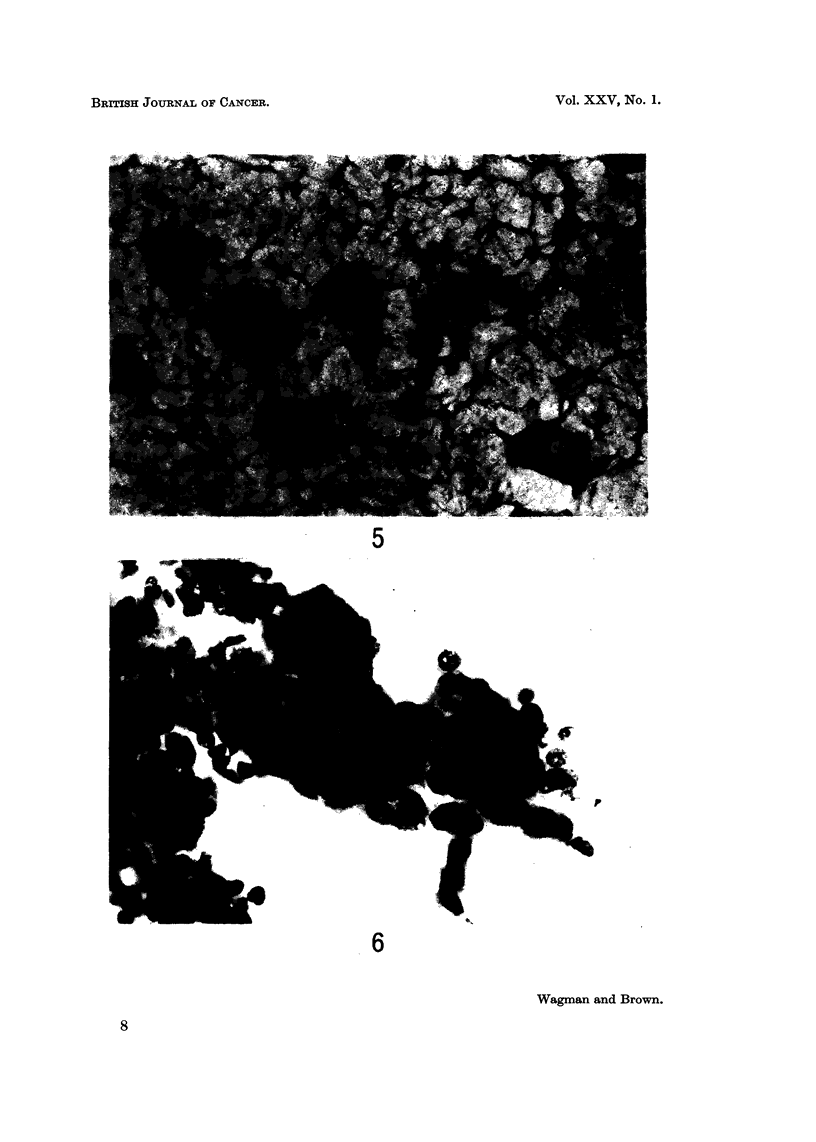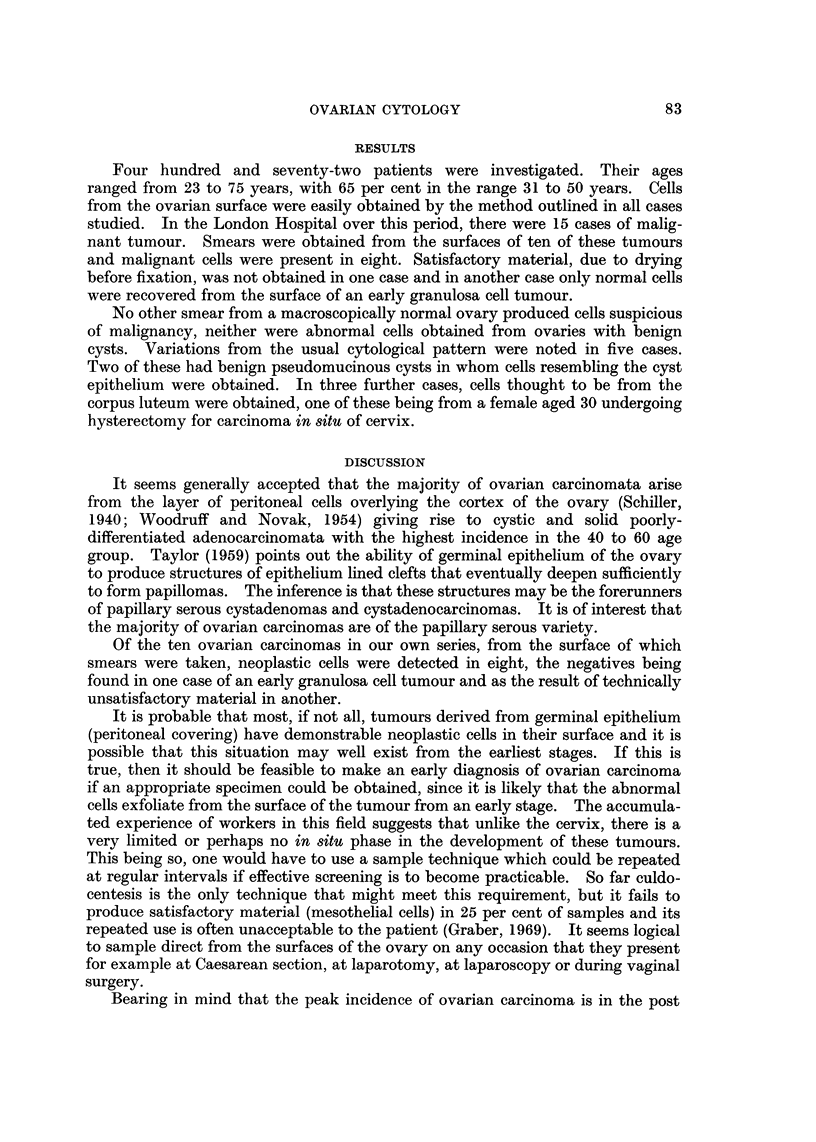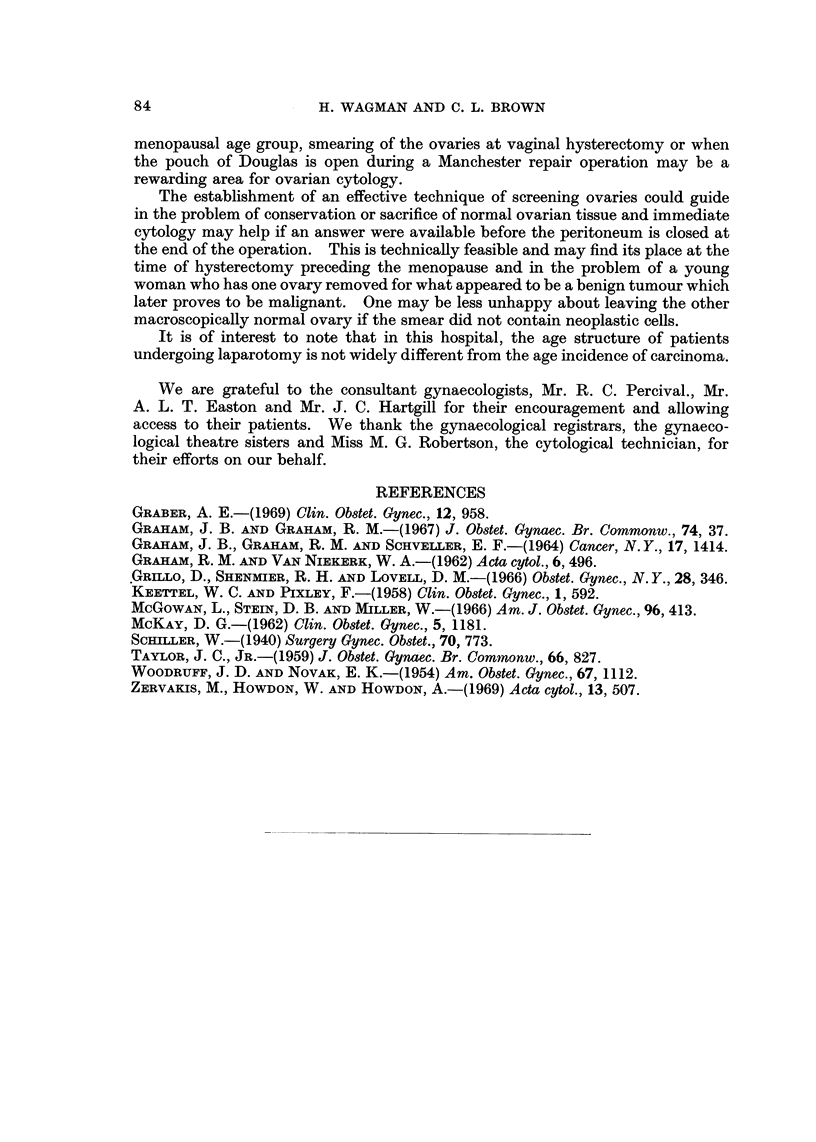# Ovarian Cytology

**DOI:** 10.1038/bjc.1971.11

**Published:** 1971-03

**Authors:** H. Wagman, C. L. Brown

## Abstract

**Images:**


### OVARIAN CYTOLOGY

### AN APPLICATION OF CYTOLOGY IN AN ATTEMPT AT THE EARLY DETECTION OF OVARIAN CARCINOMA

H. WAGMAN AND C. L. BROWN

*From the London Hospital, London, E.*1

Received for publication November 10, 1970

**SUMMARY.—The possibility of detecting pre-clinical ovarian carcinoma by ovarian cytology taken at the time of laparotomy has been studied in 472 patients. Malignant cells have been recovered from ovarian carcinomas but never from macroscopically normal ovaries. It is suggested that this simple, inexpensive technique of sampling cells from the ovarian surface should be continued to be practised on all occasions at which ovaries present such as at laparotomy or at laparoscopy, as with further experience this technique may prove to be of help in the early diagnosis of ovarian carcinoma.**

OVARIAN CARCINOMA is a serious problem to the gynaecologist. Annual deaths now exceed 3000 in England and Wales and 10,000 in the U.S.A. In England and Wales in the year 1968 it may be seen that deaths due to carcinoma of the ovary were second only to carcinoma of the breast (Table I), and it is now the leading cause of death from gynaecological malignancy.

TABLE I.—*Number of Deaths from Carcinoma of Various Sites in* 1968 (*England and Wales*)

| | |
|---|---|
| Carcinoma of breast | 10,228 |
| Carcinoma of ovary, Fallopian tube and broad ligament | 3,410 |
| Carcinoma of cervix uteri | 2,434 |
| Carcinoma of body of uterus | 1,537 |

The annual death rate is generally reported as eighteen per 100,000 of the population. It has been stated that about 30 women in 100,000 will develop ovarian cancer before the age of 45 years and that it increases to 281 per 100,000 between the ages of 45 to 60 years (Graber, 1969). The disturbing feature is that due to lack of symptoms, ovarian carcinoma does not usually present until the disease has metastasized widely, when the results of any form of treatment are likely to be poor. The 5-year survival rates are disappointing, all stages in the U.S.A. being 10·2 per cent whilst in England (London Hospital 1960–64 inclusive under 20 per cent, and these results have not significantly altered in the past 20 years.

Clinical pelvic examination is of limited value since by the time the clinician can palpate ovarian enlargement the disease may be advanced. It is already clear that vaginal cytology has little place to play in the early detection of ovarian cancer as neoplastic cells are found in only one-third of the cases of ovarian cancer (Graham and Von Niekerk, 1962).

Thus, attempts at early diagnosis assume great importance and the most notable of these is culdocentesis (Keettel and Pixley, 1958; Graham *et al.*, 1964; Graham and Graham, 1967; Grillo *et al.*, 1966; McGowan *et al.*, 1966; Zervakis *et al.*, 1969). However, the results produced by different authors are at variance; McGowan *et al.* (1966) did not find any positives in 1123 asymptomatic women, but Graham and Graham (1967) had 24 positives in 1149 asymptomatic women, two of whom had early ovarian carcinoma, two were false positives and 20 had " abnormal cellular activity ".

We have studied the effect of direct scraping of the surface of the ovary at every opportunity, usually at obstetric or gynaecological laparotomy, in an attempt to discover any potential or early ovarian carcinoma not obvious to the naked eye.

### METHOD AND MATERIAL

At the London Hospital during the period June 1968 to December 1969 inclusive, most patients undergoing laparotomy for obstetric and gynaecological indications had smears taken from the surface of the ovaries. Occasionally it was possible to smear ovaries at vaginal hysterectomy and at the time of a Manchester repair. The smears were taken as soon as possible after the peritoneal cavity was opened, whatever the naked eye appearance of the ovaries. A sterile plastic Ayres spatula was used to scrape firmly both sides of the ovarian surface. The sample obtained was smeared on to glass slides and immediately fixed in 3 per cent acetic acid in 95 per cent methanol. The firm scraping was designed to recover cells from all areas of the ovarian surface including the sulci and gyri, where one often observes a metaplasia of the peritoneal mesothelium to a cuboidal or columnar epithelium in the aging ovary (McKay, 1962). The Ayre's spatula and fixative used were chosen as simple, inexpensive pieces of equipment readily available in most gynaecological departments. The specimen was transported to the laboratory in the fixative, stained by the Papanicolaou method and screened for abnormalities by trained technicians.

---

### EXPLANATION OF PLATES

FIG. 1.—Groups of typical ovarian surface cells. The smaller, regular, darkly staining type usually form the bulk of cells obtained and are often present in large sheets. Papanicolaou stain. × 565.

FIG. 2.—The larger type of ovarian surface cell has plentiful cytoplasm and a paler staining nucleus in which, as in this group, several nucleoli may be conspicuous. Papanicolaou stain. × 565.

FIG. 3.—Cells recovered from the external surface of a benign serous cystadenoma. Their columnar shape, basal orientation of their nuclei and ciliated surface are similar to the lining cells of the tumour and distinct from the characteristics of normal ovarian surface cells. Papanicolaou stain. × 565.

FIG. 4.—In three cases proliferating granulosa cells from the corpus luteum were obtained. Numerous mitotic figures are present but the morphology of the cells is distinctive and innocent. Papanicolaou stain. × 565.

FIG. 5.—The cells with voluminous granular cytoplasm are luteinized granulosa cells obtained from a corpus luteum of pregnancy and contrast sharply with the group of normal small ovarian surface cells also shown. Papanicolaou stain. × 350.

FIG. 6.—Typical clump of carcinoma cells obtained from the external surface of a pseudomucinous cystadenocarcinoma. Note the nuclear crowding, pleomorphism and large nucleoli that are characteristic of malignant cells. Papanicolaou stain. × 565.

1

2

Wagman and Brown.

3

4

Wagman and Brown.

5

6

Wagman and Brown.

### RESULTS

Four hundred and seventy-two patients were investigated. Their ages ranged from 23 to 75 years, with 65 per cent in the range 31 to 50 years. Cells from the ovarian surface were easily obtained by the method outlined in all cases studied. In the London Hospital over this period, there were 15 cases of malignant tumour. Smears were obtained from the surfaces of ten of these tumours and malignant cells were present in eight. Satisfactory material, due to drying before fixation, was not obtained in one case and in another case only normal cells were recovered from the surface of an early granulosa cell tumour.

No other smear from a macroscopically normal ovary produced cells suspicious of malignancy, neither were abnormal cells obtained from ovaries with benign cysts. Variations from the usual cytological pattern were noted in five cases. Two of these had benign pseudomucinous cysts in whom cells resembling the cyst epithelium were obtained. In three further cases, cells thought to be from the corpus luteum were obtained, one of these being from a female aged 30 undergoing hysterectomy for carcinoma *in situ* of cervix.

### DISCUSSION

It seems generally accepted that the majority of ovarian carcinomata arise from the layer of peritoneal cells overlying the cortex of the ovary (Schiller, 1940; Woodruff and Novak, 1954) giving rise to cystic and solid poorly-differentiated adenocarcinomata with the highest incidence in the 40 to 60 age group. Taylor (1959) points out the ability of germinal epithelium of the ovary to produce structures of epithelium lined clefts that eventually deepen sufficiently to form papillomas. The inference is that these structures may be the forerunners of papillary serous cystadenomas and cystadenocarcinomas. It is of interest that the majority of ovarian carcinomas are of the papillary serous variety.

Of the ten ovarian carcinomas in our own series, from the surface of which smears were taken, neoplastic cells were detected in eight, the negatives being found in one case of an early granulosa cell tumour and as the result of technically unsatisfactory material in another.

It is probable that most, if not all, tumours derived from germinal epithelium (peritoneal covering) have demonstrable neoplastic cells in their surface and it is possible that this situation may well exist from the earliest stages. If this is true, then it should be feasible to make an early diagnosis of ovarian carcinoma if an appropriate specimen could be obtained, since it is likely that the abnormal cells exfoliate from the surface of the tumour from an early stage. The accumulated experience of workers in this field suggests that unlike the cervix, there is a very limited or perhaps no *in situ* phase in the development of these tumours. This being so, one would have to use a sample technique which could be repeated at regular intervals if effective screening is to become practicable. So far culdocentesis is the only technique that might meet this requirement, but it fails to produce satisfactory material (mesothelial cells) in 25 per cent of samples and its repeated use is often unacceptable to the patient (Graber, 1969). It seems logical to sample direct from the surfaces of the ovary on any occasion that they present for example at Caesarean section, at laparotomy, at laparoscopy or during vaginal surgery.

Bearing in mind that the peak incidence of ovarian carcinoma is in the post menopausal age group, smearing of the ovaries at vaginal hysterectomy or when the pouch of Douglas is open during a Manchester repair operation may be a rewarding area for ovarian cytology.

The establishment of an effective technique of screening ovaries could guide in the problem of conservation or sacrifice of normal ovarian tissue and immediate cytology may help if an answer were available before the peritoneum is closed at the end of the operation. This is technically feasible and may find its place at the time of hysterectomy preceding the menopause and in the problem of a young woman who has one ovary removed for what appeared to be a benign tumour which later proves to be malignant. One may be less unhappy about leaving the other macroscopically normal ovary if the smear did not contain neoplastic cells.

It is of interest to note that in this hospital, the age structure of patients undergoing laparotomy is not widely different from the age incidence of carcinoma.

We are grateful to the consultant gynaecologists, Mr. R. C. Percival., Mr. A. L. T. Easton and Mr. J. C. Hartgill for their encouragement and allowing access to their patients. We thank the gynaecological registrars, the gynaecological theatre sisters and Miss M. G. Robertson, the cytological technician, for their efforts on our behalf.

### REFERENCES

Graber, A. E.—(1969) *Clin. Obstet. Gynec.*, **12**, 958.

Graham, J. B. and Graham, R. M.—(1967) *J. Obstet. Gynaec. Br. Commonw.*, **74**, 37.

Graham, J. B., Graham, R. M. and Schveller, E. F.—(1964) *Cancer, N.Y.*, **17**, 1414.

Graham, R. M. and Van Niekerk, W. A.—(1962) *Acta cytol.*, **6**, 496.

Grillo, D., Shenmier, R. H. and Lovell, D. M.—(1966) *Obstet. Gynec., N.Y.*, **28**, 346.

Keettel, W. C. and Pixley, F.—(1958) *Clin. Obstet. Gynec.*, **1**, 592.

McGowan, L., Stein, D. B. and Miller, W.—(1966) *Am. J. Obstet. Gynec.*, **96**, 413.

McKay, D. G.—(1962) *Clin. Obstet. Gynec.*, **5**, 1181.

Schiller, W.—(1940) *Surgery Gynec. Obstet.*, **70**, 773.

Taylor, J. C., Jr.—(1959) *J. Obstet. Gynaec. Br. Commonw.*, **66**, 827.

Woodruff, J. D. and Novak, E. K.—(1954) *Am. Obstet. Gynec.*, **67**, 1112.

Zervakis, M., Howdon, W. and Howdon, A.—(1969) *Acta cytol.*, **13**, 507.